# SNAI1 is critical for the aggressiveness of prostate cancer cells with low E-cadherin

**DOI:** 10.1186/1476-4598-13-37

**Published:** 2014-02-24

**Authors:** Gagan Deep, Anil K Jain, Anand Ramteke, Harold Ting, Kavitha C Vijendra, Subhash C Gangar, Chapla Agarwal, Rajesh Agarwal

**Affiliations:** 1 Department of Pharmaceutical Sciences, Skaggs School of Pharmacy and Pharmaceutical Sciences, San Diego, USA; 2 University of Colorado Cancer Center, University of Colorado Denver, Aurora, Colorado, USA; 3 Department of Molecular Biology and Biotechnology, Tezpur University, Tezpur, India

**Keywords:** Prostate cancer, E-cadherin, SNAI1, Stemness, EMT

## Abstract

**Background:**

A better molecular understanding of prostate carcinogenesis is warranted to devise novel targeted preventive and therapeutic strategies against prostate cancer (PCA), a major cause of mortality among men. Here, we examined the role of two epithelial-to-mesenchymal transition (EMT) regulators, the adherens junction protein E-cadherin and its transcriptional repressor SNAI1, in regulating the aggressiveness of PCA cells.

**Methods:**

The growth rate of human prostate carcinoma PC3 cells with stable knock-down of E-cadherin (ShEC-PC3) and respective control cells (Sh-PC3) was compared in MTT and clonogenic assays in cell culture and in nude mouse xenograft model *in vivo*. Stemness of ShEC-PC3 and Sh-PC3 cells was analyzed in prostasphere assay. Western blotting and immunohistochemistry (IHC) were used to study protein expression changes following E-cadherin and SNAI1 knock-down. Small interfering RNA (siRNA) technique was employed to knock- down SNAI1 protein expression in ShEC-PC3 cells.

**Results:**

ShEC-PC3 cells exerted higher proliferation rate both in cell culture and in athymic nude mice compared to Sh-PC3 cells. ShEC-PC3 cells also formed larger and a significantly higher number of prostaspheres suggesting an increase in the stem cell-like population with E-cadherin knock-down. Also, ShEC-PC3 prostaspheres disintegration, in the presence of serum and attachment, generated a bigger mass of proliferating cells as compared to Sh-PC3 prostaspheres. Immunoblotting/IHC analyses showed that E-cadherin knock-down increases the expression of regulators/biomarkers for stemness (CD44, cleaved Notch1 and Egr-1) and EMT (Vimentin, pSrc-tyr416, Integrin β3, β-catenin, and NF-κB) in cell culture and xenograft tissues. The expression of several bone metastasis related molecules namely CXCR4, uPA, RANKL and RunX2 was also increased in ShEC-PC3 cells. Importantly, we observed a remarkable increase in SNAI1 expression in cytoplasmic and nuclear fractions, prostaspheres and xenograft tissues of ShEC-PC3 cells. Furthermore, SNAI1 knock-down by specific siRNA strongly inhibited the prostasphere formation, clonogenicity and invasiveness, and decreased the level of pSrc-tyr416, total Src and CD44 in ShEC-PC3 cells. Characterization of RWPE-1, WPE1-NA22, WPE1-NB14 and DU-145 cells further confirmed that low E-cadherin is associated with higher SNAI1 expression and prostasphere formation.

**Conclusions:**

Together, these results suggest that E-cadherin loss promotes SNAI1 expression that controls the aggressiveness of PCA cells.

## Background

Prostate cancer (PCA) is the most common non-cutaneous cancer, and is the second leading cause of cancer-related deaths in American men. According to the American Cancer Society, in 2013, there will be an estimated 238,590 new cases and 29,720 deaths from PCA in the United States [1]. Patients with localized PCA have a high 5-year survival rate and a relatively low mortality to incidence ratio compared to other cancer types [2]. However, in patients with clinically detectable metastasis, the median survival is reduced to only 12–15 months; therefore, metastasis is the main cause of high mortality among PCA patients [2-4]. PCA cells metastasize to several organs; however, bone is the most frequent site for metastasis [3,5]. Patients with bone metastasis suffer extreme bone pain, spinal-cord compression and fractures [6-8]. In addition, replacement of bone marrow by growing PCA cells disrupts normal haematopoiesis, causing anemia and enhanced susceptibility to infections [7]. Therefore, a better understanding of the early events associated with PCA metastasis is warranted to lower mortality and improve patient’s quality of life.

Now it is known that PCA metastasis involves multiple steps including the acquisition of invasiveness through ‘EMT’ (epithelial to mesenchymal transition), access to systemic blood or lymphatic systems (intravasation), survival in the circulation, arrest in the microvasculature and subsequent extravasation, and growth at distant organs [9]. Among these events, EMT has often been described as absolutely necessary and indispensable for metastasis [9,10]. During EMT, cancer cells shed their epithelial features, detach from epithelial sheets and undergo cytoskeletal changes towards a ‘mesenchymal phenotype’ and acquire a high degree of motility and invasiveness [10,11]. Recent studies have suggested that EMT not only enhances invasiveness and migratory potential but also confers several aggressive attributes to cancer cells such as enhanced stemness, drug and anoikis resistance, etc. [12-14]; and that these features could provide a survival advantage to cancer cells during the arduous metastasis journey from primary organs to distant metastatic sites. Therefore, understanding and targeting the role of EMT regulators in conferring an aggressive phenotype to PCA cells could be useful in effectively inhibiting metastatic progression.

The molecular regulation of EMT is extremely complex and involves numerous interconnected as well as independent pathways and signaling molecules [10,11,15]. However, several of these pathways converge together to down-regulate the expression of adherens junction molecule E-cadherin [16]. E-cadherin is a transmembrane glycoprotein that regulates cell-cell adhesion, cell polarity and shape through its interactions with E-cadherin molecules on adjacent cells as well as with the actin microfilament network via catenins (α, β and p120) [16]. The loss of E-cadherin frees catenins from the membranous pool, thus making them available for nuclear signaling, which then promote cancer cell proliferation, invasiveness and EMT [10,17]. E-cadherin expression is regulated through a combination of genetic, epigenetic, transcriptional and post-transcriptional mechanisms [10,16]. Major transcriptional repressors of E-cadherin are zinc finger family members SNAI1 (SNAIL1 in drosophila) and Slug, the basic helix-loop-helix factors E47 and Twist, and two-handed zinc factors ZEB1 and SIP1 [10]. Importantly, the loss of E-cadherin function has been implicated in the progression and metastasis of several malignancies including PCA [18,19]. Furthermore, reduced E-cadherin expression has been correlated with higher tumor grade and poor prognosis in PCA patients [20-23]. However, the molecular changes associated with E-cadherin loss that are responsible for PCA aggressiveness are still not clear. Results from the present study suggest that E-cadherin loss could enhance proliferation and stemness in PCA cells through altering the expression of several signaling molecules but mainly through its transcriptional repressor SNAI1.

SNAI1 is one of the master EMT regulators and is a promoter of metastasis, that represses the expression of several epithelial markers (E-cadherin, claudin, occludin, etc.) and enforces a mesenchymal phenotype by promoting the expression of mesenchymal genes (fibronectin, vimentin, α-SMA etc.) [10,24,25]. SNAI1 is overexpressed in several cancer cells including PCA where it is suggested to be upregulated at early stages of PCA development [26]. High SNAI1 expression in tumors often correlates with disease aggressiveness and poor prognosis [23,27,28]. SNAI1 has been implicated in cancer cell survival, cell cycle regulation, apoptosis evasion, cell adhesion, neuro-endocrine differentiation, and chemoresistance [24,25]. In the present study, we analyzed the role of SNAI1 in the aggressiveness of PCA cells with low E-cadherin expression (via Stable E-cadherin knock-down). Our results for the first time showed that SNAI1 could control the clonogenicity, stemness and invasiveness of PCA cells with low E-cadherin expression.

## Results

### E-cadherin knock-down increases proliferation of human PCA PC3 cells

**Figure 1:**
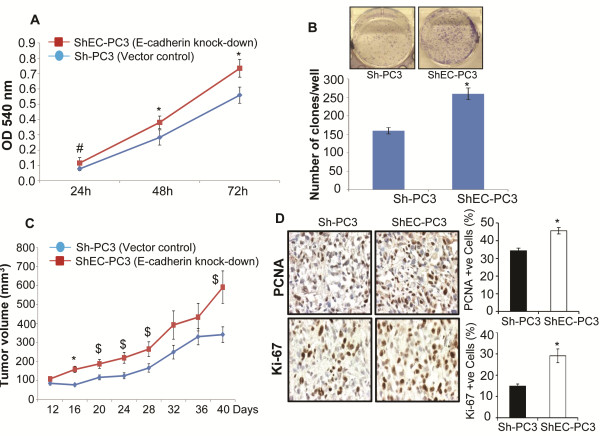
**E-cadherin knock-down increases the proliferation of human PCA PC3 cells. (A)** Multiplication rate of Sh-PC3 and ShEC-PC3 cells was determined by MTT assay. Data shown is mean ± SD of 12 samples. **(B)** Clone formation by Sh-PC3 and ShEC-PC3 cells was examined in a clonogenic assay as detailed in the methods. Number of clones with more than 50 cells were counted and presented in a bar diagram. Data shown is mean ± SD of 6 samples. **(C-D)** Sh-PC3 and ShEC-PC3 cells were injected subcutaneously in athymic nude mice, and average tumor volume (mean ± SEM) as a function of time is presented. Tumor tissues were analyzed for proliferation biomarkers (PCNA and Ki-67) by IHC. Percentage of PCNA and Ki-67 positive cells was calculated by counting the number of positive stained cells (brown stained) and the total number of cells at five arbitrarily selected fields from each tumor at 400x magnification. The data shown in the bar diagrams is the mean±SEM of 7–10 samples. *, *p* ≤ 0.001; #, *p* ≤ 0.01; $, *p* ≤ 0.05.

First we analyzed the effect of E-cadherin loss on the proliferation of PC3 cells where ShEC-PC3 cells with E-cadherin knock-down showed higher proliferation at 24, 48, and 72 hrs after seeding compared to vector control Sh-PC3 cells (Figure 1A). MTT assay results were further confirmed in a clonogenic assay which showed that E-cadherin knockdown significantly (*p* ≤ 0.001) enhanced the clonogenicity of PC3 cells (Figure 1B). Next, ShEC-PC3 and Sh-PC3 cells were subcutaneously injected in athymic male nude mice to compare their *in vivo* growth rate. As shown in Figure 1C, both Sh-PC3 and ShEC-PC3 cells formed xenografts, however, the tumor volume was consistently higher in ShEC-PC3 cells compared to Sh-PC3 cells. At the end of the study, xenograft tissues were analyzed for proliferation biomarkers (PCNA and Ki-67) by IHC. ShEC-PC3 tumors showed a higher expression of both PCNA and Ki-67 positive cells (Figure 1D), suggesting an increased proliferation rate *in vivo*. Taken together, these results suggested that E-cadherin knock-down increases the proliferation rate of PC3 cells both *in vitro* and *in vivo*.

### E-cadherin knock-down enhances the stemness of human PCA PC3 cells

**Figure 2:**
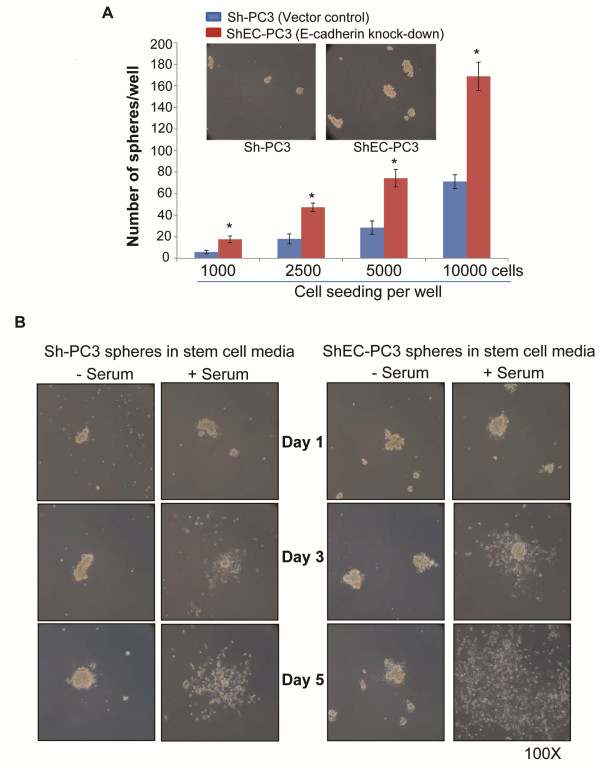
**E-cadherin knock-down enhances the stemness of human PCA PC3 cells. (A-B)** Sh-PC3 or ShEC-PC3 cells were plated on 6 well Corning ultra-low attachment plates in DMEM/F-12(Ham) media containing supplements B27 and N2. Prostasphere formation was measured after 5 days. Thereafter, prostaspheres were collected and plated on normal cell culture plate with or without serum and monitored for 5 days. Representative pictures are shown for prostaspheres’ state at day 1, day 3 and day 5. Data shown is mean ± SD of 3–6 samples. * *p* ≤ 0.001.

Next we examined the effect of E-cadherin knock-down on the stemness of PCA cells in a prostasphere assay. The prostasphere assay is considered the ‘gold standard’ to determine the self-renewal capability of a stem-like cell population (CSC) in cell culture [29-31]. This assay is based upon the principle that only CSC can survive and grow without attachment in the absence of serum. As shown in Figure 2A, ShEC-PC3 cells formed a significantly (p ≤ 0.001) higher number and bigger sized prostaspheres compared to Sh-PC3 cells. These results suggested that E-cadherin knock-down increases the stemness in PC3 cells.

We also analyzed the disintegration or differentiation of prostaspheres in the presence of attachment with or without the addition of serum. Prostaspheres were pipetted and re-plated on normal attachment culture plates with or without 10% FBS. In the absence of serum, both ShEC-PC3 and Sh-PC3 prostaspheres attached to the bottom of the plate but their disintegration was hardly visible even after 5 days. However, in the presence of serum both Sh-PC3 and ShEC-PC3 cells disintegrated into bulk of growing cells; though, in general, ShEC-PC3 prostaspheres generated a bigger mass of growing bulk cells compared to Sh-PC3 prostaspheres (Figure 2B).

### E-cadherin knock-down increases the expression of stemness, EMT, and bone metastasis biomarkers in human PCA PC3 cells both *in vitro* and *in vivo*

**Figure 3:**
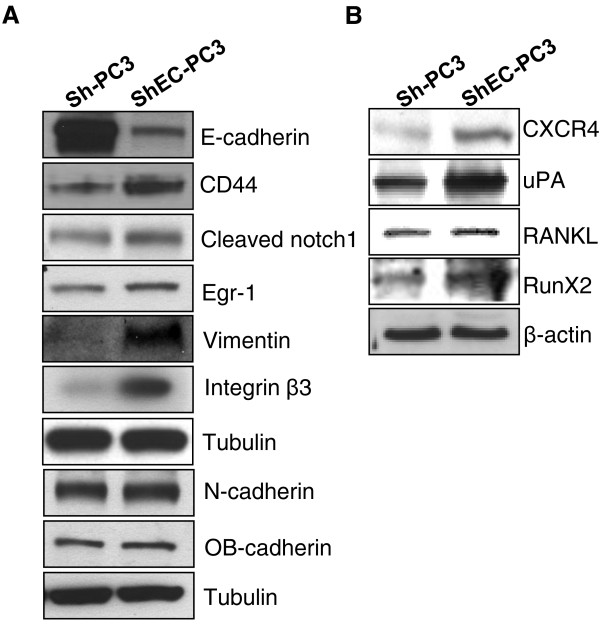
**E-cadherin knock-down increases the expression of stemness, EMT, and bone metastasis biomarkers in PC3 cells*. *(A-B)** Sh-PC3 or ShEC-PC3 cells were collected at similar confluency and total cell lysates were prepared and analyzed for the protein expression of E-cadherin, CD44, cleaved Notch-1, Egr-1, Vimentin, Integrin β3, N-cadherin, OB-cadherin, CXCR4, uPA, RANKL, and RunX2. Tubulin and β-actin were used as loading controls.

**Figure 4:**
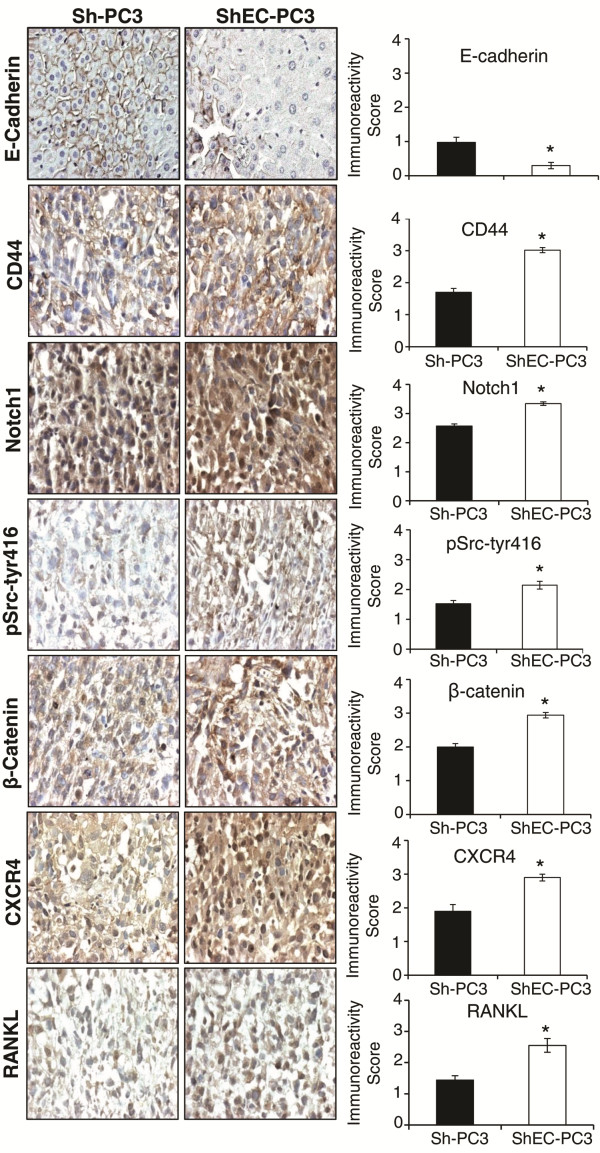
**Expression of stemness, EMT, and bone metastasis biomarkers in Sh-PC3 and ShEC-PC3 xenograft tissues*.*** Sh-PC3 and ShEC-PC3 xenograft tissues were analyzed for the expression of E-cadherin, CD44, Notch1, pSrc-tyr416, β-catenin, CXCR4 and RANKL by IHC as detailed in the methods. Immunoreactivity was analyzed in 5 random areas for each tumor tissue and was scored as 0+ (no staining), 1+ (weak staining), 2+ (moderate staining), 3+ (strong staining), 4+ (very strong staining). IHC scores (as mean ± SEM) are shown as bar diagram of 5–10 samples.

Next, we analyzed the effect of E-cadherin knock down on stemness and mesenchymal biomarkers in human PCA PC3 cells. Western blot analysis showed that E-cadherin knock-down resulted in increased expression of CD44 and cleaved Notch1 in ShEC-PC3 cells (Figure 3A), which are well known biomarkers for stemness [32-34]. E-cadherin knock-down also increased Egr-1 expression (Figure 3A), which is a regulator of CD44 promoter activity [35]. Furthermore, E-cadherin knock-down resulted in a strong increase in EMT biomarkers, the intermediate filament protein Vimentin and Integrin β3 expression (Figure 3A). However, E-cadherin knock-down resulted in only a slight or no significant increase in the expression of other cadherins namely N-cadherin and OB-cadherin (Figure 3A). Besides, we have earlier reported a strong increase in the levels of phosphorylated pSrc-tyr416 following E-cadherin knock-down in PC3 cells [36], a kinase associated with increased PCA invasiveness and bone metastasis [37]. E-cadherin knock-down also increased the expression of several other proteins that are important in bone metastasis. As shown in Figure 3B, ShEC-PC3 cells showed higher expression of CXCR4, which is known to play an important role in the migration of PCA cells towards the chemotactic signal (SDF1α) secreted by bone endothelial cells [8,38]. We also observed increased expression of uPA, RANKL and RunX2, which are considered important for initiating/promoting osteoclastogenesis in bone by PCA cells (Figure 3B) [7,8]. Next, we examined the expression of the above mentioned biomarkers in ShEC-PC3 and Sh-PC3 tissues from the xenograft experiment (Figure 1C). As shown in Figure 4, ShEC-PC3 xenograft tissues showed low E-cadherin level but exhibited significantly higher expression of CD44, Notch1, pSrc-tyr416, β-catenin, CXCR4 and RANKL compared to Sh-PC3 xenograft tissues.

### E-cadherin knock-down increases SNAI1 expression in human PCA PC3 cells both *in vitro* and *in vivo*

**Figure 5:**
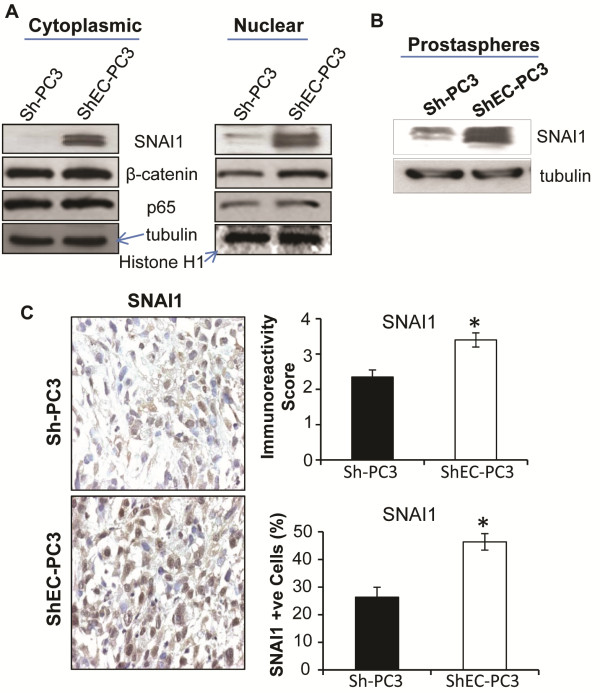
**Effect of E-cadherin knock-down on the expression of SNAI1 and other transcriptional factors. (A)** Sh-PC3 and ShEC-PC3 cells were collected at similar confluency and nuclear/cytoplasmic fractions were prepared and analyzed for SNAI1, β-catenin, and p65 expression by Western blotting. Tubulin and histone H1 were used as loading control for cytoplasmic and nuclear fractions respectively. **(B)** Sh-PC3 and ShEC-PC3 prostaspheres were collected following centrifugation and cell lysates were prepared and analyzed for SNAI1 expression by Western blotting. **(C)** Sh-PC3 and ShEC-PC3 xenograft tissues were analyzed for the expression of SNAI1 by IHC. Immunoreactivity score was analyzed in 5 random areas for each tumor tissue and was scored as 0+ (no staining), 1+ (weak staining), 2+ (moderate staining), 3+ (strong staining), 4+ (very strong staining). Percentage of SNAI1 positive cells was calculated by counting the number of positive stained cells (brown stained) and the total number of cells at five arbitrarily selected fields from each tumor at 400x magnification. The data shown in the bar diagrams is the mean±SEM of 7–10 samples. *, *p* ≤ 0.001.

Next, we examined the expression of several transcriptional factors (SNAI1, β-catenin, and NF-κB) in Sh-PC3 and ShEC-PC3 cells. We observed a strong increase in the expression of SNAI1 in both cytoplasmic and nuclear fractions of ShEC-PC3 cells compared to Sh-PC3 cells (Figure 5A). However, we observed only a modest increase in nuclear β-catenin and a slight increase in nuclear NF-κB subunit p65 expression without significant changes in the cytoplasmic β-catenin and p65 expression between ShEC-PC3 and Sh-PC3 cells (Figure 5A). Importantly, we also observed a strong increase in SNAI1 expression in the prostaspheres formed by ShEC-PC3 cells compared to Sh-PC3 cells (Figure 5B). Furthermore, SNAI1 expression was also increased in ShEC-PC3 xenograft tissues both in terms of increase in the overall immunoreactivity score as well as the percentage of SNAI1-positive cells (Figure 5C).

### SNAI1 is critical for the stemness, clonogenicity and invasiveness of ShEC-PC3 cells

**Figure 6:**
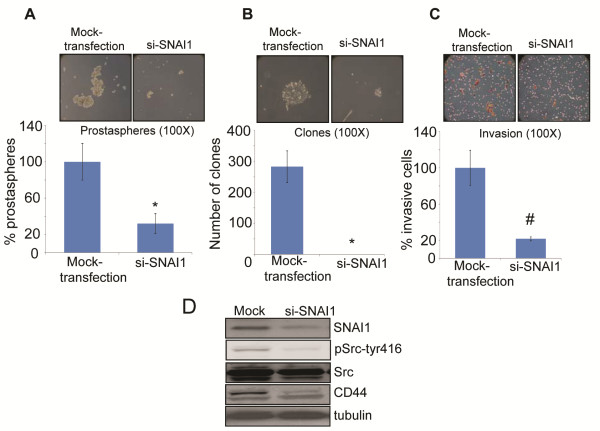
**SNAI1 knock-down inhibited the prostaspheres formation, clonogenicity, and invasiveness of ShEC-PC3 cells. (A-C)** SNAI1 expression was knocked down using SNAI1 specific siRNA. Mock and si-SNAI1 transfected cells were collected and analyzed for prostasphere formation, clonogenicity and invasiveness. Representative photomicrographs are shown at 100x. **(D)** Total cell lysates were prepared from mock and si-SNAI1 transfected ShEC-PC3 cells, and analyzed for SNAI1, pSrc-tyr416, Src, and CD44 by Western blotting. Tubulin was used as loading control. *, *p* ≤ 0.001; #, *p* ≤ 0.01.

Since we observed a strong increase in SNAI1 expression following E-cadherin knock-down, we next examined whether the increase in SNAI1 controls the stemness, clonogenicity and invasiveness of ShEC-PC3 cells. Accordingly, we knocked-down SNAI1 expression in ShEC-PC3 cells using SNAI1 specific siRNA and performed prostasphere, clonogenic and invasion assays. As shown in Figure 6A-6C, SNAI1 knock-down strongly decreased the number as well as size of prostaspheres and clones (≥50 cells) (*p* ≤ 0.001). SNAI1 knock-down also compromised the invasiveness of ShEC-PC3 cells (*p* ≤ 0.001). Furthermore, SNAI1 knock-down resulted in decreased pSrc-tyr416, Src and CD44 levels, suggesting a role for SNAI1 in regulating their expression. These results confirmed the central role of SNAI1 in controlling stemness, clonogenicity, and invasiveness in ShEC-PC3 cells.

### Low E-cadherin is associated with high SNAI1 and prostasphere formation

**Figure 7:**
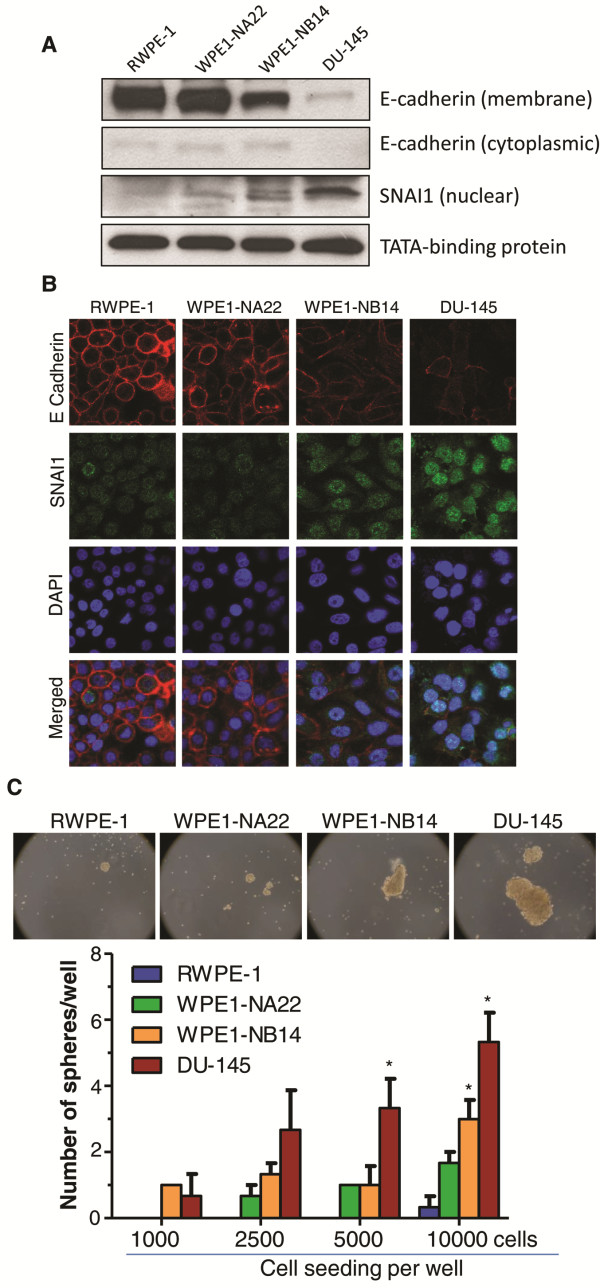
**Low E-cadherin is associated with high SNAI1 and prostasphere formation. (A-B)** E-cadherin and SNAI1 expression was analyzed in RWPE-1, WPE1-NA22, WPE1-NB14, and DU-145 cells via immunoblotting and confocal microscopy methods. Representative confocal pictures are shown (at 1500x) where Alexa Fluor 555-red is for E-cadherin, Alexa Fluor 488-green is for SNAI1, while DAPI-blue stains nuclei. **(C)** RWPE-1, WPE1-NA22, WPE1-NB14, and DU-145 cells were plated on 6 well Corning ultra-low attachment plates in DMEM/F-12(Ham) media containing supplements B27 and N2. Prostasphere formation was measured after 8 days. Representative prostasphere pictures are shown at 100x. Data shown is mean ± SEM of 3 samples. * *p* ≤ 0.001 (compared to RWPE-1 prostasphere number).

Next, we employed 4 cell lines (RWPE-1, WPE1-NA22, WPE1-NB14 and DU-145) and compared their E-cadherin and SNAI1 expression as well as capability to form prostaspheres. RWPE-1 is a non-tumorigenic HPV18 immortalized cell line derived from peripheral zone of an adult human prostate [39]. WPE1-NA22 and WPE1-NB14 were derived from RWPE-1 following 50 and 100 μg/ml MNU (N-methyl-N-nirtosourea) exposure, respectively [39]. These cell lines have been well characterized [39], and WPE1-NB14 cells are considered more aggressive than WPE1-NA22 cells in terms of their proliferation, invasiveness and xenograft formation *in vivo*[39]. DU-145 is an androgen-independent human PCA cell line derived from brain metastasis. Together, these cell lines represent various stage of PCA development i.e. from normal to advanced metastatic stage. Immunoblot analysis showed low E-cadherin expression in the membrane fraction of DU-145 and WPE1-NB14 cells compared with WPE1-NA22 and RWPE-1 cells (Figure 7A). Relatively low E-cadherin was observed in cytoplasmic fraction of all the cell lines tested with least expression in DU-145 cells (Figure 7A). On the contrary, nuclear SNAI1 expression was highest in DU-145 cells followed by WPE1-NB14, WPE1-NA22 and RWPE-1 cells (Figure 7A). Immunoblotting results for E-cadherin and SNAI1 expression in these 4 cell lines were further confirmed by confocal microscopy (Figure 7B). Immunofluorescence analysis also showed that RWPE-1 cells have polygonal morphology with intact cell-cell contact that was progressively lost in WPE1-NA22, WPE1-NB14 and DU-145 cells together with a decrease in E-cadherin and an increase in SNAI1 expression (Figure 7B). Next, we compared the prostasphere formation in these 4 cell lines. As shown in Figure 7C, RWPE-1 and WPE1-NA22 cells did not form prostaspheres or formed relatively smaller sized prostaspheres, while WPE1-NB14 and DU-145 cells formed larger number and bigger sized prostaspheres. Overall, DU-145 cells (with lowest E-cadherin and highest SNAI1 expression) formed highest number and biggest sized prostaspheres among all the four cell lines studied here (Figure 7C).

## Discussion

Lately, targeted therapies are being exploited to develop personalized medicines based upon the specific mutations and molecular alterations in cancer cells. Accordingly, the identification and functional characterization of important early molecular alterations, which are involved in the growth and progression of prostate cancer (PCA), remain vital towards devising novel targeted preventive and therapeutic strategies. In PCA patients, the main cause of death is the metastatic spread of the disease; however, it remains extremely difficult to predict indolent versus aggressive tumor when diagnosed at an early stage. Now, EMT has been suggested to be required by stationery cancer cells to acquire phenotypic and functional characteristics for metastasis. Therefore, EMT biomarkers have been extensively examined to predict disease outcome [20-23,26]. In this regard, E-cadherin loss or reduced expression at the membrane of neoplastic cells has often been associated with worsening histological grade and clinical stage along with poor prognosis in a variety of cancers including prostate, gastric, and breast [18,19,23,40,41]. However, heterogeneity in E-cadherin expression has been observed in PCA metastatic tissues with few studies reporting reduced E-cadherin expression while others reporting normal or higher E-cadherin expression in metastatic tissues compared to primary tumor tissues [21,22,42-45]. Putzke *et al.* even reported difference in the E-cadherin expression dependent upon the metastatic organ site with significantly higher E-cadherin expression observed in bone metastatic tissues compared to soft tissue metastases [41]. The expression of another EMT regulator i.e. SNAI1 has also been correlated with an increased risk of tumor relapse and poor survival in breast cancer patients, and with the progression of colorectal cancer [23,46,47]. Recently, Whiteland *et al.*[23], using 215 archival PCA patient tissue samples analyzed the expression and sub-cellular localization of several EMT biomarkers to correlate them with disease outcome. This study revealed that loss of E-cadherin expression at the cellular membrane of PCA cells is significantly associated with increasing Gleason score and clinical stage, and a poor survival. Furthermore, nuclear SNAI1 expression was significantly increased in PCA tissue and was strongly associated with increasing Gleason score and clinical stage but did not demonstrate a significant association with PSA (prostate specific antigen) recurrence or patient survival. Therefore, E-cadherin and SNAI1 are important in the clinical progression of the disease; and in the present study, we demonstrate the role of E-cadherin and SNAI1 in conferring several aggressive characteristics to PCA cells such as higher proliferation rate, clonogenicity, stemness and increased expression of biomarkers for stemness, EMT, and metastasis.

There have been several studies suggesting that EMT not only enhances the motility and invasiveness of cancer cells, but also provide several additional aggressive features such as stemness, therapeutic and anoikis resistance etc. Gupta *et al.*[13] have shown that E-cadherin down-regulation not only induces EMT but also enhances the CSC population in breast cancer cells. In fact, a greater degree of overlapping has been observed in the CSC population as well as invasive or metastatic cells. Balic *et al.* reported that most of the early disseminated cancer cells detected in the bone marrow of breast cancer patients have a putative CSC phenotype [48]. In another study, Aktas *et al.* showed that a major proportion of circulating tumor cells in the blood of breast cancer patients has stem cell characteristics [49]. One explanation put forward to describe high-stemness in metastatic cancer cells is that ‘stationary CSC’ could undergo EMT and give rise to ‘metastatic CSC’ [50-52]. Another line of experimental evidence suggests that EMT induction in differentiated neoplastic epithelial cells (non-CSC population) not only enhances invasiveness but also their stemness [13,14,51,53,54]. In any case, increased stemness might provide the necessary plasticity to cancer cells required to adapt to varying microenvironments during the arduous metastatic journey and colonization at distant organ sites. Results from the present study also support the argument that ‘EMT enhances stemness’ as E-cadherin knock-down significantly enhanced the clone and prostasphere formation by PC3 cells. However, Celia-Terrassa *et al.* have reported that PC3-derived clonal populations enriched for epithelial phenotype exhibit a stronger expression of self-renewal/pluripotency gene networks and more aggressive attributes [55]. Furthermore, the suppression of epithelial program inhibited the self-renewal/pluripotency gene network of tumor cells, their capacity to grow under attachment-independent conditions, and their tumorigenic and metastatic potentials [55]. This study also suggested the coexistence of heterogeneous populations with epithelial or mesenchymal phenotype interacting and co-operating to impact on the tumor’s potency for local invasiveness and distant metastasis. Together, these studies highlight the plasticity in PCA cells where epithelial, mesenchymal, and intermediate or a mix of these states could impart contextual advantages dependent upon cancer stage and/or tumor microenvironment.

SNAI1 is a member of the zinc-finger transcription factor family and is known to repress E-cadherin expression [56]. SNAI1 is located on chromosome 20q13 that exhibits gene amplification in tumor samples from metastatic PCA [57]. Increased SNAI1 expression is considered an early event in the progress of prostate carcinogenesis but is limited to cells with invasive properties [26]. SNAI1 is also reported to enhance RANKL expression, osteoclastogenesis and bone colonization [58]. Furthermore, SNAI1 regulates CSC activity and tumorigenicity in breast and colorectal carcinoma cells [14,28]; and CRC patients with abundant SNAI1 expression exhibit high metastasis [28]. Baygi *et al.* reported that SNAI1 knock-down significantly reduced the viability of human PCA cells and prevented their re-attachment potential through modulating the expression of Integrins [24]. This study also suggested that SNAI1 primarily acts as a survival factor and inhibitor of cellular senescence. SNAI1 overexpression in ARCaP PCA cells induced EMT through ROS (reactive oxygen species) generation, increase in the expression of inflammatory chemokine CCL5 and ERK activation [59]; and SNAI1 knock-down in C4-2 and ARCaP cells overexpressing SNAI1 significantly compromised their migration potential [60]. Neal *et al.* have reported that higher SNAI1 expression could promote migration and invasion in PCA cells through negatively regulating the expression of protease inhibitor Maspin [61]. SNAI1 has also been reported to increase the expression of mesenchymal markers Vimentin and Fibronectin as well as other proteins involved in cancer invasion such as metalloproteinases 2 and 9, and various transcription factors such as ZEB-1 and LEF-1 [62,63]. SNAI1 expression is inversely correlated with RKIP (Raf kinase inhibitor protein), a metastatic suppressor protein that inhibits cell survival, proliferation and invasiveness through targeting Raf-1/MEK/ERK and NF-κB signaling pathways [63,64]. In the present study, we observed that knock-down of E-cadherin expression in PC3 cells resulted in a strong increase in SNAI1 expression both in cell culture (cells and prostaspheres) as well as xenograft tissues; and that SNAI1 inhibition reduced the stemness, clonogenicity and invasiveness of ShEC-PC3 cells. It is possible that SNAI1 inhibition reduces the survival of ShEC-PC3 cells potentially by inducing senescence and/or apoptosis involving down-regulation of Integrins, Vimentin or other EMT regulators, decrease in ROS level, and increase in Maspin and/or RKIP as reported in above studies. Our results also suggested that SNAI1 inhibition could reduce the stemness of ShEC-PC3 cells through a decrease in CD44 expression (as shown in Figure 6D). Also, SNAI1 knock-down in ShEC-PC3 cells could reduce the invasiveness through inhibiting Src phosphorylation (Figure 6D). Therefore, there could be several molecular mechanisms possible for the inhibitory effect of SNAI1 knock-down on the stemness and invasiveness of ShEC-PC3 cells, and these need to be investigated further in future.

It is now well established that SNAI1 transcriptionally down-regulates E-cadherin expression; however, here we report an interesting finding that SNAI1 expression is increased following E-cadherin knock-down in PC3 cells. Therefore, the loss of E-cadherin and SNAI1 up-regulation could be inter-related during prostate carcinogenesis, where SNAI1 increase could repress E-cadherin expression, and vice versa. Earlier studies have shown that GSK-3β (glycogen synthase kinase-3 beta) phosphorylates SNAI1 and promotes its export from the nucleus and subsequent degradation by the proteasome in the cytosol [19,65]. Conversely, PAK1 (p21-activated kinase) could phosphorylate SNAI1 to promote its nuclear localization and activity as a transcriptional factor [19,66]. Du *et al.* reported that protein kinase D1 (PKD1) could also phosphorylate SNAI1 at Ser11, triggering its nuclear export via 14-3-3σ binding [19]. Wu *et al.* have shown that NF-κB also plays an important role in the stabilization of SNAI1 [19,67]. One possibility for the observed increase in SNAI1 expression with E-cadherin knock-down could be increased nuclear β-catenin which could enhance SNAI1 expression. Similarly, there was a slight increase in nuclear p65 expression with E-cadherin knock-down, which could also enhance SNAI1 expression. Also, E-cadherin knock-down could modify the phosphorylation status of SNAI1 favoring its nuclear localization and stabilization possibly through a decrease in GSK-3β and/or PKD1 or an increase in PAK1 and/or NF-κB activity. Further studies are warranted to clearly define the molecular mechanisms through which E-cadherin loss results in higher SNAI1 expression.

Together, the existing literature as well as results from the present study suggest that it is feasible to prevent metastasis in PCA patients with localized disease through re-activating/increasing E-cadherin expression or through targeting SNAI1 expression in PCA cells by using existing or novel cancer preventive agents [68-70]. For example, earlier we have reported that in TRAMP (transgenic adenocarcinoma of the mouse prostate) mice E-cadherin expression is lost while SNAI1 expression is increased with disease progression from PIN to poorly differentiated adenocarcinoma stages [70]; and the administration of the cancer chemopreventive agent Silibinin, a natural flavonoid from Milk thistle extract, strongly enhanced E-cadherin expression while it decreased SNAI1 expression and prevented PCA metastasis to distant organs [70]. Recently, Harney *et al.* developed a novel strategy to target SNAI1 expression in cancer cells [71]. They conjugated Co(III)Schiff base complexes with specific oligonucleotide i.e. Co(III)-Ebox selectively targeting the E-box-binding zinc finger family transcriptional factors resulting in enhanced E-cadherin promoter activity in MCF7 cells [71]. But it should be cautioned that SNAI1 plays an important role during embryonic development and is also considered an important stem cell regulator, therefore SNAI1 inhibitors should be specifically targeted towards cancer cells. Also, SNAI1 inhibition could possibly cause the re-expression of E-cadherin as well as other epithelial markers in metastatic tissues, where higher E-cadherin or epithelial characteristics could favor better survival and proliferation [41,55]. This clearly reflects the challenge of understanding and targeting the epithelial plasticity in PCA, as E-cadherin promotion and SNAI1 downregulation could prevent growth and invasiveness in primary tumors; however, could potentially enhance growth at certain metastatic sites.

## Conclusions

Overall, results from the present study suggest that the EMT regulators- E-cadherin and SNAI1 could be used for disease prognosis as well as suitably targeted to prevent PCA metastatic progression.

## Methods

### Cells culture and reagents

Human prostate carcinoma PC3, RWPE-1, WPE1-NA22, WPE1-NB14 and DU-145 cells were obtained from American Type Culture Collection (Manassas, VA). Sh-PC3 and ShEC-PC3 cells were cultured in RPMI1640 medium supplemented with 10% heat inactivated fetal bovine serum (FBS), 100 U/ml penicillin G, 100 μg/ml streptomycin sulfate and puromycin at 37°C in a humidified 5% CO_2_ incubator. RWPE-1, WPE1-NA22, and WPE1-NB14 cells were cultured in keratinocyte serum-free medium containing 50 μg/ml bovine pituitary extract and 5 ng/ml epidermal growth factor. DU-145 cells were cultured in RPMI1640 medium supplemented with 10% heat inactivated FBS, 100 U/ml penicillin G and 100 μg/ml streptomycin sulfate. Media and other cell culture materials as well as fluorescently conjugated anti-mouse and anti-rabbit IgG antibodies were from Invitrogen Corporation (Gaithersburg, MD). Antibodies for β-catenin, Vimentin, Egr-1 (early growth response-1), CXCR4, uPA (Urokinase plasminogen activator), RANKL (receptor activator of nuclear factor kappa-B ligand), p65, RunX2, Histone H1 and E-cadherin shRNA plasmid were purchased from Santa Cruz Biotechnology (Santa Cruz, CA). Antibodies for E-cadherin, CD44, Integrin β3, SNAI1, cleaved Notch1, pSrc-tyr416, total Src, and anti-rabbit peroxidase-conjugated secondary antibody were obtained from Cell Signaling (Beverly, MA). SNAI1, N-cadherin, OB-cadherin and TATA-binding protein (TBP) antibodies were from Abcam (Cambridge, MA). Puromycin, DAPI (4′,6-diamidino-2-phenylindole), and β-actin antibody were from Sigma-Aldrich (St Louis, MO). ECL detection system and anti-mouse HRP-conjugated secondary antibody were from GE Healthcare (Buckinghamshire, UK). On-Target plus smart pool SNAI1 siRNA was purchased from Thermo Scientific (Waltham, MA) and HiPerfect transfection reagent was from Qiagen (Valencia, CA). Antibody for α-tubulin was from Lab Vision Corporation (Fremont, CA). All other reagents were obtained in their commercially available highest purity grade.

### Transfection

PC3 cells with stable knock-down of E-cadherin (ShEC-PC3 cells) and respective control cells (Sh-PC3 cells) were generated as published earlier [36]. For SNAI1 knock-down, ShEC-PC3 cells (~5×10^5^) were plated in 60 mm dishes for 24 hrs. SNAI1 siRNA and transfection reagents were mixed in 100 μl serum free media and added drop-wise over ShEC-PC3 cells. Serum containing media was added 1 hr after transfection. Cells were collected after 48 hrs and knock-down was confirmed by Western blotting. In other studies, cells were also collected and analyzed in clonogenic, prostasphere and invasion assays.

### MTT assay

Sh-PC3 and ShEC-PC3 Cells were plated at a density of 1000 cells/well in 96-well plate under standard culture conditions. At the end of indicated time-point, fresh media containing 20 μl of MTT (5 mg/ml stock) was added, and incubated for another 4 h in a CO2 incubator. At the end, media was removed and 200 μl of DMSO was added to each well. Color intensity was measured by taking absorbance at 540 nm.

### Clonogenic assay

Sh-PC3 and ShEC-PC3 cells (~ 1×10^3^ per well) were plated in 6-well plates. Fresh media was added every 48 h. At the end of the 7^th^ day, cells were washed twice with ice cold PBS, fixed with a mixture of methanol and glacial acetic acid (3:1) for 10 minutes and then stained with 1% crystal violet in methanol for 15 minutes followed by washing with deionized water. Colonies with more than 50 cells were scored and counted under the microscope. Photomicrographs were taken using Canon Power Shot digital camera.

### Matrigel invasion assay

Invasion assay was performed using matrigel invasion chambers from BD Biosciences as per vendor’s protocol. Briefly, the bottom chambers were filled with RPMI1640 media with 10% FBS and the top chambers (inserts) were seeded with 50,000 cells (mock or SNAI1 siRNA transfected) per well in RPMI1640 media (with 0.5% FBS). Top chambers have a thin layer of matrigel, and PCA cells invaded through the matrigel layer and 8 micron membrane pores. After 22 h of incubation under standard culture conditions, cells on the top matrigel surface (non-invasive cells) were scraped with a cotton swab and the cells spreading on the bottom sides of the membrane (invasive cells) were fixed, stained, and mounted. Images were captured using Cannon Power Shot A640 camera on Zeiss inverted microscope and total number of invasive cells was counted and percentage of cell invasion was calculated.

### Prostasphere assay

Sh-PC3, ShEC-PC3, RWPE-1, WPE1-NA22, WPE1-NB14 and DU-145 cells (1000, 2500, 5000, or 10000 cells) were plated in 6 well Corning ultra-low attachment plates in DMEM/F-12(Ham) media containing supplements B27 and N2 (from Invitrogen). Cell culture was monitored daily to assess that sphere originated from single cell; however cells or spheres aggregation cannot be completely ruled out. In each case, number of prostaspheres (with average diameter more than 75 μm) formed after 5–8 days was counted under a microscope. Prostasphere images were captured using Cannon Power Shot A640 camera on Zeiss inverted microscope.

### Immunoblotting

Total or nuclear/cytoplasmic lysates were prepared following published protocol [72,73] and sub-cellular fractionations were prepared as per vendor’s protocol (ThermoFisher Scientific, Rockford, IL). Approximately, 50–70 μg of protein lysate per sample was denatured in 2x sample buffer and subjected to sodium dodecyl sulfate–polyacrylamide gel electrophoresis (SDS-PAGE) on 6 or 12% Tris–glycine gel (as required based upon the protein molecular weight). The separated proteins were transferred on to nitrocellulose membrane followed by blocking with 5% non-fat milk powder (w/v) in Tris-buffered saline (10 mM Tris–HCl, pH 7.5, 100 mM NaCl, 0.1% Tween 20) for 1 h at room temperature. Membranes were probed for the protein levels of desired molecules using specific primary antibodies followed by the appropriate peroxidase-conjugated secondary antibody and visualized by ECL detection system. To ensure equal protein loading, each membrane was stripped and re-probed with appropriate loading control. The autoradiograms/bands were scanned with Adobe Photoshop 6.0 (Adobe Systems, San Jose, CA). In each case, blots were subjected to multiple exposures on the film to make sure that the band density is in the linear range.

### Xenograft study and Immunohistochemistry (IHC)

Athymic (*nu/nu*) male nude mice were housed at the University of Colorado Denver (UCD) animal care facility. Protocols were approved by UCD Institutional Animal Care and Use Committee. Approximately, 1 million Sh-PC3 or ShEC-PC3 cells were suspended in 0.05 ml of serum-free medium (RPMI1640), mixed with 0.05 ml of matrigel and were s.c. injected in each flank of male athymic nude mouse (NCI-Frederick, Bethesda, MD) (n = 5 with total 10 xenografts for each group) to initiate tumor growth. Once the tumor xenograft started growing, their sizes were measured (every 4^th^ day) in two dimensions using a digital caliper. The tumor volume was calculated by the formula: 0.5236 L_1_(L_2_)^2^, where L_1_ is the long diameter and L_2_ is short diameter. At the end, each tumor was carefully dissected and processed for IHC following published methods [74]. Briefly, sections were incubated with desired primary antibody followed by incubation with a specific biotinylated secondary antibody, followed by conjugated HRP streptavidin, DAB working solution, and finally counterstained with hematoxylin. Stained tumor sections were analyzed by Zeiss Axioscope 2 microscope and images were captured by the AxioCam MrC5 camera at 400x magnifications.

### Confocal imaging

RWPE-1, WPE1-NA22, WPE1-NB14 and DU-145 cells were grown on cover slips and incubated in media for 24 hrs. Cells were then fixed in 3.7% formaldehyde, washed with PBS, permeabilized with 0.2% Triton X-100 overnight at 4°C along with primary antibodies for E-cadherin and SNAI1. Cells were then washed with PBS and incubated with secondary antibodies and DAPI for 60 min. Cell images were captured at 1500× magnification on a Nikon inverted confocal microscope using 561/488/405 nm laser wavelengths to detect E-cadherin (Red), SNAI1 (Green) and DAPI (Blue) emissions, respectively.

### Statistical analysis

Statistical analysis was performed using SigmaStat 2.03 software (Jandel Scientific, San Rafael, CA). Data was analyzed using one way ANOVA and a statistically significant difference was considered at *p* < 0.05.

## Competing interests

The authors declare that they have no competing interests

## Authors’ contributions

GD contributed to the conception and design of the study, coordinated the research, performed the xenograft, Western blotting, prostasphere and knock-down experiments, and wrote the manuscript. AKJ performed the IHC. AR and HT performed the confocal studies, Western blotting for several proteins and proof read the manuscript. KCV and SCG carried out the MTT and clonogenic assays. CA contributed in the design of several experiments, Western blotting and proof read the manuscript. RA conceived the study, elaborated its design, coordinated the research, and critically revised the manuscript. All the authors have read and approved the final manuscript.
